# Doing Nicely

**Published:** 1916-10

**Authors:** 


### BUFFALO MEDICAL JOURNAL

**A Monthly Review of Medicine and Surgery**

EDITOR AND PUBLISHER, DR. A. L. BENEDICT, 228 Summer St., corner of Elmwood Ave., Buffalo, (Address for all communications. Please make personal and telephone calls before 1 P. M.)

Third, in age, on the western continent. Independent, but supports professional organizations. News limited mainly to Western N. Y. and Penn.

Subscription $2.00 a year; $3.00 for two years in advance. Changes and errors in address or failure or duplication of delivery, should be reported immediately.

Yearly Volume 72 OCTOBER, 1916 Number 3

### "Doing Nicely"

The Medical Economist, August, tells of a man who telephoned daily to inquire of a certain hospital, about the progress of his daughter, who had infantile paralysis. He received the above reply or some equivalent, each time. Shortly after the last inquiry, he received a notification that his daughter was dying—and at an entirely different hospital.

If our telephone is ever suddenly discontinued, those who try to reach us may conclude with reasonable probability, that some sweet voiced nurse has told us that a patient—and we naturally do not inquire when we have no proper interest in the case—was "doing nicely," and that the company has removed our instrument because of our remarks. Up to date, we have succeeded in keeping within the bounds of language allowed but we cannot answer for the future. Even the laity are getting to the point where they realize just what "doing nicely" means and those who do not, are very apt to become rude and troublesome when they realize that the speaker or the authorities that govern her reply, have not the slightest idea of giving accurate information about a case or of identifying it in any way. And they may become still more rude and troublesome when some such information as the Medical Economist mentions, falsifies what they considered an accurate and consoling bulletin of the patient's progress.

It is both the right and the duty of a hospital to guard professional secrets. Reasonable care should be taken to identify a physician or relative or other representative of a patient. Mutual convenience renders it desirable that this care should not be so thorough as to eliminate the telephone entirely for the purpose of securing information of a patient's progress. Common sense dictates that such information should be somewhat more technical in replying to a physician than to a member of the laity. But, one of the courses should be followed: either information should be refused, or it should be given in good faith and accurately.

